# Reactive oxygen species mediate growth and death in submerged plants

**DOI:** 10.3389/fpls.2013.00179

**Published:** 2013-06-04

**Authors:** Bianka Steffens, Anja Steffen-Heins, Margret Sauter

**Affiliations:** ^1^Plant Developmental Biology and Plant Physiology, Kiel UniversityKiel, Germany; ^2^Micro- and Nanostructures in Food, Kiel UniversityKiel, Germany

**Keywords:** reactive oxygen species, adventitious root growth, epidermal cell death, aerenchyma formation, ROS detection, electron paramagnetic resonance spectroscopy, ethylene

## Abstract

Aquatic and semi-aquatic plants are well adapted to survive partial or complete submergence which is commonly accompanied by oxygen deprivation. The gaseous hormone ethylene controls a number of adaptive responses to submergence including adventitious root growth and aerenchyma formation. Reactive oxygen species (ROS) act as signaling intermediates in ethylene-controlled submergence adaptation and possibly also independent of ethylene. ROS levels are controlled by synthesis, enzymatic metabolism, and non-enzymatic scavenging. While the actors are by and large known, we still have to learn about altered ROS at the subcellular level and how they are brought about, and the signaling cascades that trigger a specific response. This review briefly summarizes our knowledge on the contribution of ROS to submergence adaptation and describes spectrophotometrical, histochemical, and live cell imaging detection methods that have been used to study changes in ROS abundance. Electron paramagnetic resonance (EPR) spectroscopy is introduced as a method that allows identification and quantification of specific ROS in cell compartments. The use of advanced technologies such as EPR spectroscopy will be necessary to untangle the intricate and partially interwoven signaling networks of ethylene and ROS.

## INTRODUCTION

In aerobic cells about 1% of metabolically consumed O_2_ goes into reactive oxygen species (ROS) generation (Puntarulo et al., 1988). ROS are generated from molecular oxygen by a number of reductive steps. Superoxide anions ($O_{2}^{•–}$), hydroxyl radical (^•^OH), singlet oxygen (^1^O_2_), hydroperoxyl radical (${HO}_{2}^{•}$), and ozone (O_3_) are generated by a one-electron to three-electron reduction of oxygen with reductive power being provided by electron carriers in mitochondria and chloroplasts (Blokhina and Fagerstedt, 2010a; Chang et al., 2012; reviewed in Shapiguzov et al., 2012). Hydrogen peroxide (H_2_O_2_) is a non-radical that can cross membranes by diffusion and it can be transported by specific aquaporins (Bowler et al., 1992; Bienert et al., 2007; Borisova et al., 2012). H_2_O_2_ is produced by a two-electron reduction of molecular oxygen catalyzed by the respiratory burst NADPH oxidase (RBOH) at the plasma membrane. RBOH proteins in plants are homologs of NADPH oxidase 2 of mammals (Torres et al., 1998) and belong to the cytochrome *b* family. H_2_O_2_ can also be produced spontaneously by dismutation of either $O_{2}^{•–}$ or ${HO}_{2}^{•}$.

**FIGURE 1:**
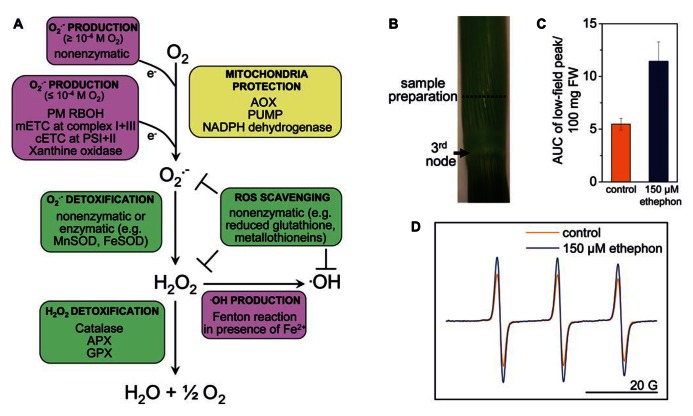
**Reactive oxygen species homeostasis in plants.** **(A)** ROS are generated enzymatically or non-enzymatically depending on internal O_2_ levels. In photosynthetic tissues, the main sources of ROS are the chloroplasts and peroxisomes (reviewed in Shapiguzov et al., 2012). In photosynthetically active chloroplasts, $O_{2}^{•–}$ is generated at photosystems I and II. In the dark, at low light conditions or in photosynthetically inactive tissues ROS are produced mainly in mitochondria. $O_{2}^{•–}$ is produced by the flavin mononucleotide binding subunit of NADH dehydrogenase in complex I and by ubiquinol-cytochrome *bc1* reductase in complex III of the mitochondrial electron transport chain (mETC), or by RBOH at the plasma membrane. $O_{2}^{•–}$ is dismutated to H_2_O_2_ non-enzymatically, by a manganese-containing isoform of the superoxide dismutase (MnSOD) in the matrix (Møller, 2001), or by FeSOD in chloroplasts and Cu/ZnSOD in the cytosol. ^•^OH is a highly reactive ROS that is produced in the Fenton reaction from H_2_O_2_ in the presence of metals such as Fe^2^^+^. The uncoupling protein (PUMP), the alternative oxidase (AOX) and an alternative NAD(P)H dehydrogenase protect mitochondria from unwarranted ROS production. Antioxidants such as reduced glutathione, metallothioneins; and enzymes such as superoxide dismutase (SOD), catalase, ascorbate peroxidase (APX), and glutathione peroxidase (GPX) exist in different cellular compartments to detoxify ROS. **(B)** Stem sections of deepwater rice cultivar Pin Gaew 56 were treated with 150 μM ethephon or left untreated as a control for 20 h. Internodal tissue was collected 10 mm above the third node for EPR analysis. **(C)** The double integral of the low-field peak (AUC, area under peak) was determined to compare ROS levels detected with the spin probe TMT-H (2,2,6,6-tetramethylpiperidinium) in control sections and after ethephon treatment. **(D)** Typical EPR spectra of the spin probe after the reaction with ROS. Two hundred microliters of a 1-mM spin probe solution were added to 100 mg of tissue and incubated for 10 min. EPR measurements were performed on a Bruker Elexsys E500 spectrometer at room temperature with the following settings: center field 3513 G, sweep width 65 G, microwave frequency 9.84 GHz, microwave power 2 mW, modulation amplitude 1.2 G, conversion time 20.01 ms, time constant 40.96 ms (A. Steffen-Heins, B. Steffens, unpublished).

Protection of mitochondria from unwarranted ROS production is provided by the alternative oxidase (AOX) and by an alternative type II, non-proton-pumping, Ca^2^^+^-dependent NADPH dehydrogenase (ND; reviewed in Blokhina and Fagerstedt, 2010b). While AOX and ND protect mitochondria from oxidative stress the oxidized state of intermediates of the electron transport chain at the same time results in a decrease in ATP synthesis (Borecký, 2006). Antioxidant activity is provided throughout the cell by low molecular mass components such as reduced glutathione, reduced ascorbic acid, tocopherols, tannins, ubiquinol, and phenolic compounds, and by ROS scavenging enzymes such as superoxide dismutase (SOD), catalase (CAT), ascorbate peroxidase (APX), and glutathione peroxidase (GPX). Non-enzymatic ROS scavenging proteins such as thioredoxin and metallothioneins also contribute to ROS homeostasis. The type of ROS that accumulates is ultimately determined by the balance between ROS producing and ROS scavenging activities. For instance SOD determines the rate of H_2_O_2_ production and CAT the rate of H_2_O_2_ metabolism. A change in either activity affects H_2_O_2_ steady-state levels. Generation and detoxification mechanisms of the main ROS are summarized in **Figure 1A**. This review focuses on ROS as signaling intermediates in submergence adaptation and it summarizes methods used to identify the ROS involved.

## SUBMERGENCE-INDUCED AND ROS-MEDIATED GROWTH AND CELL DEATH RESPONSES

As explained above, the balance between production and scavenging of ROS controls cellular ROS levels in plants. Oxidative stress occurs when these processes are imbalanced. High light, heat, pathogen invasion, wounding, low oxygen, and re-aeration after a phase of low oxygen stress increase ROS generation while low light conditions that arise for example during submergence decrease ROS production (Suzuki et al., 2012; Szarka et al., 2012). ROS are generated via enzymatic as well as non-enzymatic reactions. Which of the two mechanisms take place is influenced by the cellular oxygen concentration. Non-enzymatic one-electron O_2_ reduction occurs at 10^-^^4^ M and higher concentrations of O_2_ while enzymatic reactions take place at lower oxygen concentrations. The same holds true for mitochondrial electron transport and respiration establishing a link between oxygen concentration, mitochondrial ATP production, and oxidative stress.

Soil water logging and partial or complete submergence limit gas diffusion which results on one hand in oxygen shortage and on the other hand in the accumulation of the volatile hormone ethylene in flooded tissues. In rice, ethylene promotes adventitious root growth, death of epidermal cells overlaying adventitious root primordia, and parenchymal cell death which results in aerenchyma formation. All of these responses are mediated by ROS. In deepwater rice, ethylene-induced adventitious root growth is abolished when RBOH activity is inhibited indicating that root growth in response to flooding is controlled by ROS that are generated at the plasma membrane (Steffens et al., 2012). RBOH activity is regulated by small G proteins (Baxter-Burrell et al., 2002; Wong et al., 2007). Inhibition of CAT enhances internal ROS levels and results in growth promotion revealing that either superoxide anion or H_2_O_2_ are the active ROS. Scavenging of H_2_O_2_ by potassium iodide partially reduces ethylene-dependent root growth supporting this finding.

Epidermal cells that overlay adventitious root primordia at the stem node of rice plants undergo cell death prior to the emergence of the adventitious root. Epidermal cell death is induced by ethylene which promotes cell death via H_2_O_2_ (Steffens and Sauter, 2009). The metallothionein MT2b is a non-enzymatic H_2_O_2_ scavenger in rice. Genetic downregulation of *MT2b* elevates endogenous ROS levels in rice cells (Wong et al., 2004). In epidermal cells that undergo cell death *MT2b* is downregulated by ethylene suggesting that ethylene promotes ROS accumulation and hence cell death induction via MT2b. In fact, constitutive genetic downregulation of *MT2b* enhances epidermal cell death constitutively showing that modulation of ROS scavenging by MT2b is sufficient to alter cell death rates (Steffens and Sauter, 2009). *MT2b* is downregulated in epidermal cells overlaying adventitious roots not only by ethylene but also by H_2_O_2_ itself revealing a feedback loop that autoamplifies H_2_O_2_ accumulation. While induction of adventitious root growth by ethylene is also promoted by ROS, downregulation of *MT2b* does not alter root growth rate suggesting that regulation of epidermal cell death and of adventitious root growth rely on different ROS signaling pathways.

Aside from the formation of adventitious roots, the development of internal gas spaces by way of programmed cell death is another major adaptation that helps plants to cope with flooding stress. Aerenchyma are constitutively formed in deepwater and lowland rice stems and leaf sheaths. Aerenchyma formation is enhanced in internodes of deepwater rice by ethylene which promotes formation of $O_{2}^{•–}$ (Steffens et al., 2011). In lowland rice varieties aerenchyma formation in leaf sheaths is increased upon submergence (Parlanti et al., 2011). In the lowland rice variety FR13A, the ETHYLENE RESPONSE FACTOR (ERF) SUBMERGENCE 1A (SUB1A) is induced by ethylene during submergence and suppresses ethylene biosynthesis by feedback inhibition (Fukao et al., 2006; Xu et al., 2006). In FR13A, ROS accumulate independent of ethylene signaling but are none the less responsible for submergence-induced aerenchyma formation in leaf sheaths (Parlanti et al., 2011). The lowland rice variety Arborio Precoce does not possess *SUB1A* and ROS do not accumulate during leaf sheath aerenchyma formation. However, Parlanti et al. (2011) postulate that an early transient ROS accumulation that occurs prior to ethylene signaling promotes aerenchyma formation. Hence, aerenchyma formation in response to submergence appears to be controlled by ROS in lowland and deepwater rice varieties. In some but not all varieties ROS accumulation is controlled by ethylene signaling which may influence the timing of cell death induction. In conclusion, ROS are central regulators of plant adaptation to submergence.

## ROS HOMEOSTASIS AND SIGNALING IN HYPOXIC PLANTS

At low oxygen conditions, ROS production in *Arabidopsis* occurs predominantly at the plasma membrane through RBOH and in mitochondria. *RbohD* one of the 10 RBOH genes of *Arabidopsis* is induced at low oxygen (Pucciariello et al., 2012). Activation of RBOH occurs furthermore at the protein level by small G proteins such as ROP in *Arabidopsis* (Baxter-Burrell et al., 2002) and OsRac1 in rice (Wong et al., 2007). In mitochondria $O_{2}^{•–}$, ^•^OH, ^1^O_2_, ${HO}_{2}^{•}$, and O_3_ are generated as a result of an overreduction of the redox chain during anoxia (Chang et al., 2012). In *Arabidopsis*, ROS originating in mitochondria activate the mitogen-activated protein kinase MAPK6 to improve survival at hypoxic conditions (Chang et al., 2012). In plant mitochondria, the AOX transfers four electrons from ubiquinone to oxygen thereby preventing ROS production from an overreduced ubiquinone pool (Umbach et al., 2005). AOX is encoded by five genes of the multigene families AOX1 and AOX2 in *Arabidopsis* (Considine et al., 2002; Borecký, 2006). Constitutive activation of AOX in *Arabidopsis* or overexpression of *Arabidopsis* *AOX1a* in tobacco decreases mitochondrial ROS production (Maxwell et al., 1999) while inhibition of AOX increases ROS production (Maxwell et al., 1999; Umbach et al., 2005). In barley roots, AOX activity is elevated at anoxic conditions (Skutnik and Rychter, 2009). Detoxification of ROS serves to prevent oxidative damage but at the same time may alter a ROS signal. Future work is required to consolidate or distinguish between the two pathways.

The dismutation of $O_{2}^{•–}$ to H_2_O_2_ is mediated by FeSOD in chloroplasts, MnSOD in mitochondria, and by Cu/ZnSOD in chloroplasts and in the cytoplasm. The enzymatic reaction is 10,000-fold faster than spontaneous dismutation. H_2_O_2_ is detoxified to H_2_O and O_2_ by CAT. In addition, soluble, extracellular, or cell wall-associated peroxidases detoxify H_2_O_2_. Peroxidases also generate $O_{2}^{•–}$ and H_2_O_2_ (Mika et al., 2010). Anoxia and hypoxia increase SOD activity in wheat and *Iris pseudacorus* (Monk et al., 1987; Biemelt et al., 1998) but not in barley roots (Szal et al., 2004) while in maize flooded for 7 days $O_{2}^{•–}$ levels increase due to reduced SOD activity possibly pointing to a regulatory role. In the wetland species *Alternanthera philoxeroides* and *Hemarthria altissima*, SOD and CAT activities are differentially regulated during flooding depending on the survival strategy (Luo et al., 2012). In *Alternanthera philoxeroides* that shows the “escape” strategy (Bailey-Serres and Voesenek, 2008), SOD and CAT activities are downregulated in leaves but recover after de-submergence. *H. altissima* pursues a “quiescence” strategy and displays high SOD and CAT activities in submerged leaves. This differential response is compatible with the view that ROS contribute to shoot growth control.

Lipoxygenases catalyze the hydroperoxidation of poly-unsaturated fatty acids. In wheat roots and in corn leaves levels of $O_{2}^{•–}$ and H_2_O_2_ increase after re-aeration resulting in elevated lipid peroxidation and loss of membrane integrity (Albrecht and Wiedenroth, 1994). Lipoxygenase activity in anoxia-treated potato cells correlates with the duration of the low oxygen treatment (Pavelic et al., 2000). Lipids are protected from oxidative damage by tocopherols and tocotrienols known as vitamin E. Anoxia-intolerant *I. germanica* has more β-tocopherol as compared to anoxia-tolerant *I. pseudacorus* while α-tocopherol content does not differ (Blokhina et al., 2000). Anoxia induces tocopherol deprivation in both *Iris* species. However, the decline in tolerant *I. pseudacorus* sets in later than in *I. germanica* possibly contributing to the observed tolerance (Blokhina et al., 2000). Along the same line, the submergence-tolerant rice variety FR13A protects lipids during submergence while the anoxia-sensitive variety CT6241 displays enhanced lipid peroxidation (Santosa et al., 2007). The protective mechanism of FR13A is, however, not understood.

In conclusion, regulation of ROS levels in flooded plants relies on the regulation of ROS producing and ROS scavenging mechanisms. It is not always clear if changes in ROS levels exclusively cause or prevent damage or if and how they contribute to signaling. What has become clear, however, is that ROS abundance is regulated at different levels in different plant species. Mechanisms of ROS regulation are numerous and have not been fully analyzed in any one species or been compared stringently between flooding-resistant and flooding-prone ecotypes. This should be achieved in future research to identify unifying mechanisms that characterize flooding-resistant plants. The following paragraph summarizes and comments on methods currently used to detect ROS.

## DETECTION OF ROS BY SPECTROPHOTOMETRICAL AND STAINING METHODS

It is challenging to monitor ROS abundance in plant cells due to their low concentration and short half-life. For example, ^•^OH has a half-life of a few nanoseconds and $O_{2}^{•–}$ of tenths of microseconds. Another challenge is the spatial resolution as ROS can accumulate in different cell compartments. Detection must be sensitive and specific for defined ROS. Indirect measurement of ROS generation is possible by analyzing lipid peroxidation of unsaturated fatty acids in membranes. This method was used to investigate ROS production under low oxygen stress in oat and wheat roots, Iris rhizomes, and rice seedlings (Blokhina et al., 1999; Santosa et al., 2007), and after re-aeration in rice (Fukao et al., 2011). Methods commonly used for ROS detection are summarized in **Table 1** and described below.

**Table 1:** Common ROS detection methods.

**ROS**	**ROS detection method**	**Condition/plant species**	**Reference**
	**Spectrophotometrical methods**		
$O_{2}^{•–}$	Irreversible oxidation of epinephrine	Hypoxia, barley roots	Szal et al. (2004)
		Submergence, rice internodes	Steffens et al. (2011)
	Cleavage of 4-MUF-glu	Oxidative stress, *Arabidopsis*	Kush and Sabapathy (2001)
H_2_O_2_	Oxidation of Amplex Red	Hypoxia and anoxia, wheat roots	Biemelt et al. (2000)
		Hypoxia and anoxia, *Arabidopsis* seedlings	Pucciariello et al. (2012)
		Submergence, rice	Parlanti et al. (2011)
	**Histochemical approaches**		
$O_{2}^{•–}$	Oxidation of NBT	Submergence, rice leaves	Fukao et al. (2011)
		Submergence, rice internodes	Steffens et al. (2011)
		Submergence, adventitious roots	Steffens et al. (2012)
		Submergence, nodal epidermis	Steffens and Sauter (2009)
H_2_O_2_	Cerium perhydroxide	HR, lettuce cells	Bestwick et al. (1997)
	Oxidation of DAB	Submergence, *Alternanthera philoxeroides*, *H. altissima*	Luo etal. (2012)
		Submergence, rice leaves	Fukao et al. (2011)
		Submergence, rice internodes	Steffens et al. (2011)
		Submergence, adventitious roots	Steffens et al. (2012)
		Submergence, nodal epidermis	Steffens and Sauter (2009)
	**Live cell imaging**		
ROS/RNS	DCFH_2_-DA	Submergence, rice leaves	Parlanti et al. (2011)
		*Arabidopsis* roots	Chang et al. (2012)
		*Arabidopsis* leaves	Umbach et al. (2012)
H_2_O_2_	Amplex red	Tobacco leaves	Snyrychová et al. (2009)
^1^O_2_	Singlet Oxygen Sensor Green	Wounding, *Arabidopsis* leaves	Flors et al. (2006)

Short-lived $O_{2}^{•–}$ were measured by irreversible oxidation of epinephrine to adrenochrome (Chance et al., 1979) in hypoxic barley roots (Szal et al., 2004) and during ethylene-induced aerenchyma formation in rice stems (Steffens et al., 2011). In cell cultures, $O_{2}^{•–}$ concentration was determined by 4-methyl-beta-D-umbelliferyl glucopyranoside (4-MUF-glu) in a fluorometric assay (Kush and Sabapathy, 2001) to analyze a role of the annexin-like protein Oxy5 from *Arabidopsis* in the oxidative stress response. 4-MUF-glu is cleaved by $O_{2}^{•–}$ to the fluorescent form 4-methylumbelliferone (4-MUF). A common disadvantage of spectrophotometrical methods is the relatively high demand for biological material.

Hydrogen peroxide can be quantified by recording the oxidation of *N*-acetyl-3,7-dihydroxyphenoxazine (Amplex Red), a derivative of dihydro-resorufin in the presence of horseradish peroxidase in an assay that uses plant tissue extract. During the reaction, Amplex Red is converted to the fluorescent resorufin. Amplex Red was used to analyze H_2_O_2_ production in hypoxic and anoxic wheat roots (Biemelt et al., 2000), in hypoxic and anoxic *Arabidopsis* seedlings (Pucciariello et al., 2012), and to compare differences in H_2_O_2_ production in two rice cultivars after 3 days of submergence (Parlanti et al., 2011). This probe is useful for *in planta* studies as it is membrane-permeable. This assay does, however, not provide spatial resolution.

Cell type-specific ROS detection is possible with histochemical approaches. Cerium chloride (CeCl_2_) or 3,3′-diaminobenzidine (DAB, Bestwick et al., 1997; Thordal-Christensen et al., 1997; Blokhina et al., 2001) are useful to visualize H_2_O_2_. In the presence of CeCl_2_, H_2_O_2_ produces stable precipitates of cerium perhydroxides with higher electron density that can be observed by transmission electron microscopy. Localization and quantification of H_2_O_2_ in different cell compartments is possible. DAB reacts with H_2_O_2_ in a peroxidase-catalyzed reaction resulting in an oxidized insoluble brown precipitate. For the microscopic detection of $O_{2}^{•–}$, the nitro-substituted aromatic compound nitroblue tetrazolium (NBT) is useful. Oxidized NBT forms precipitates resulting in a blue staining. Detection of H_2_O_2_ and $O_{2}^{•–}$ at the cellular level was used to analyze ROS accumulation during submergence in rice leaves (Fukao et al., 2011), ethylene-induced and ROS-mediated epidermal and parenchymal cell death in rice, and adventitious root growth in rice (Steffens and Sauter, 2009; Steffens et al., 2011, 2012). These precipitation techniques usually require removal of chlorophyll and are hence not suited for live cell imaging.

Fluorescent probes such as derivates of dichloro-dihydrofluorescein diacetate can non-destructively detect ROS through live cell imaging. The probes permeate membranes in the non-fluorescent uncharged forms and are kept in the charged form in the cytosol, or in organelles after cleavage of the acetate groups by esterases (Kristiansen et al., 2009). Green fluorescence develops due to oxidation of the ROS-reactive charged form by $O_{2}^{•–}$ or H_2_O_2_ but also by peroxyl radical (ROO^•^) and peroxynitrite (ONOO^-^; Tarpey and Fridovich, 2001). The fluorescent probe 2′-7′-dichloro-dihydrofluorescein diacetate (DCFH_2_-DA) was used for ROS and reactive nitrogen species (RNS) detection in leaf sheath sections of submerged rice seedlings (Parlanti et al., 2011), in roots of *Arabidopsis* seedlings (Chang et al., 2012), and in *Arabidopsis* leaves (Umbach et al., 2012) through confocal laser scanning microscopy. Amplex Red can also be used for specific detection of H_2_O_2_ in cells by confocal laser scanning microscopy (Snyrychová et al., 2009). For ^1^O_2_ detection the fluorescent dye Singlet Oxygen Sensor Green was used to monitor wound-induced production of this highly reactive ROS in *Arabidopsis* leaves (Flors et al., 2006).

Reactive oxygen species likely play an even more important role in the regulation of developmental events than has been recognized so far. The methods presented here will be important in unraveling this role.

## ROS DETECTION WITH ELECTRON PARAMAGNETIC RESONANCE SPECTROSCOPY – A SENSITIVE TECHNIQUE TO ANALYZE ROS *IN PLANTA*

A sensitive technique to identify, quantify and visualize short-lived ROS is electron paramagnetic resonance (EPR) spectroscopy. ROS are detected by EPR using spin traps or spin probes with different properties including lipophilicity, reaction kinetic and stability of adducts. Spin traps including the nitrones DMPO (5,5-dimethyl-pyrroline-N-oxide) and its phosphorylated analog DEPMPO (5-diethoxyphosphoryl-5-methyl-1-pyrroline-N-oxide) are diamagnetic and form stable adducts with transient radicals to transform them into longer-lived radical species (Bavčić, 2005). Suitable spin traps are defined by either the ability to exclusively trap one ROS as was shown for EMPO (5-ethoxycarbonyl-5-methyl-pyrroline-N-oxide) and BMPO (5-*tert*-butoxycarbonyl-5-methyl-1-pyrroline-N-oxide) that specifically detect $O_{2}^{•–}$ (Bavčić, 2008) or to lead to different specific signature EPR spectra. Improved spin traps like DEPMPO have a longer lifetime than DMPO-adducts, reduced degradation of the spin adducts and a faster reaction kinetic leading to a sufficient trapping of $O_{2}^{•–}$ and ^•^OH. 4-POBN [α(4-pyridyl-1-oxide)-N-tert-butylnitrone] detects specifically ^•^OH and has been used to analyze radicals in the medium surrounding growing maize roots (Liszkay et al., 2004) and in growing cucumber and *Arabidopsis* roots (Renew et al., 2005). In addition, specific EPR spectra of ^•^OH were obtained from defined cucumber root zones (Renew et al., 2005) suggesting that this technique allows for spatial resolution of ROS detection.

Spin probes can be used either as endogenous nitroxides that are reduced by ROS to the EPR-silent hydroxylamines or *vice versa.* Endogenous cyclic hydroxylamines are oxidized by ROS to EPR-active nitroxides. The very fast reaction rates between ROS and hydroxylamine are a major advantage compared with spin traps. The efficiency of hydroxylamines to detect $O_{2}^{•–}$ is very high so that very low concentrations of the hydroxylamines are necessary to detect $O_{2}^{•–}$ (1 mM compared with 10–50 mM used in spin traps), and side effects can be minimized (Dikalov et al., 2011). This is mainly due to the high reactivity of radicals so that their reaction site is very close to their generation site (Heins et al., 2007). Since the reaction of hydroxylamines toward ROS is unspecific, ROS must be identified by alternative approaches. Additions of scavengers of defined ROS such as SOD are useful (Dikalov et al., 2011). The spin probe technique has been used to measure $O_{2}^{•–}$ in *Arabidopsis* roots (Warwar et al., 2011) and in thylakoid membranes (Kozuleva et al., 2011; Borisova et al., 2012). Using the spin probe technique we showed that ethylene enhances ROS levels in rice internodes possibly related to parenchymal cell death and aerenchyma formation (**Figures 1B–D**; Steffens et al., 2011). EPR spectroscopy may turn out as a useful tool to analyze ROS in defined cells and to evaluate their contribution to submergence adaptation.

## Conflict of Interest Statement

The authors declare that the research was conducted in the absence of any commercial or financial relationships that could be construed as a potential conflict of interest.
